# The Effect of Heart Rate Variability Biofeedback Training on Mental Health of Pregnant and Non-Pregnant Women: A Randomized Controlled Trial

**DOI:** 10.3390/ijerph16061051

**Published:** 2019-03-23

**Authors:** Judith Esi van der Zwan, Anja C. Huizink, Paul M. Lehrer, Hans M. Koot, Wieke de Vente

**Affiliations:** 1 Department of Clinical, Neuro- and Developmental Psychology, Amsterdam Public Health Research Institute, Vrije Universiteit Amsterdam, Van der Boechorststraat 7, 1081BT Amsterdam, The Netherlands; j.e.vanderzwan@amc.uva.nl (J.E.v.d.Z.); j.m.koot@vu.nl (H.M.K.); 2 Department of Psychiatry, Rutgers—Robert Wood Johnson Medical School, Piscataway, 671 Hoes Lane, Piscataway, NJ 08854, USA; lehrer@rwjms.rutgers.edu; 3 Research Institute of Child Development and Education, and Research Priority Area Yield, University of Amsterdam, Nieuwe Achtergracht 127, 1001NG Amsterdam, The Netherlands; w.devente@uva.nl

**Keywords:** stress, anxiety, depression, sleep, psychological well-being, HRV-biofeedback

## Abstract

In this study, we examined the efficacy of heart rate variability (HRV)-biofeedback on stress and stress-related mental health problems in women. Furthermore, we examined whether the efficacy differed between pregnant and non-pregnant women. Fifty women (20 pregnant, 30 non-pregnant; mean age 31.6, *SD* = 5.9) were randomized into an intervention (*n* = 29) or a waitlist condition (*n* = 21). All participants completed questionnaires on stress, anxiety, depressive symptoms, sleep, and psychological well-being on three occasions with 6-week intervals. Women in the intervention condition received HRV-biofeedback training between assessment 1 and 2, and women in the waitlist condition received the intervention between assessment 2 and 3. The intervention consisted of a 5-week HRV-biofeedback training program with weekly 60–90 min. sessions and daily exercises at home. Results indicated a statistically significant beneficial effect of HRV-biofeedback on psychological well-being for all women, and an additional statistically significant beneficial effect on anxiety complaints for pregnant women. No significant effect was found for the other stress-related complaints. These findings support the use of HRV-biofeedback as a stress-reducing technique among women reporting stress and related complaints in clinical practice to improve their well-being. Furthermore, it supports the use of this technique for reducing anxiety during pregnancy.

## 1. Introduction

Stress-related mental health problems are common in Western societies, and include high levels of stress, anxiety, or depression [1,2]. These mental health problems are increasingly contributing to high societal costs [3] through healthcare costs and sick-leave from work. For instance, the European Agency for Safety and Health at Work estimated the total costs of work-related stress and psychosocial risks to be 240 billion euros per year for Europe [4]. Besides the societal costs, suffering from stress-related mental health problems may lead to poor general well-being and eventually burnout [5] and psychiatric disorders such as major depression and anxiety disorders [6,7]. Furthermore, stress can lead to poor physical health such as a dysregulated immune system and cardiovascular problems [8,9]. Thus, effective stress-reducing methods are of utmost importance. One promising and easy-to-learn method to reduce stress is heart rate variability (HRV)-biofeedback [10].

The goal of HRV-biofeedback is to increase HRV through paced breathing exercises (~6 breaths per minute). In order to do this, a small hand-held device measures the user’s heart rate, and the device uses this information to give feedback on the optimal breathing frequency. Advantages of this technique include the ease at which it can be learned and the fact that it does not require straining physical exercise. Higher HRV is associated with the ability to self-regulate emotionally [11,12]. HRV-biofeedback has shown to be effective in reducing stress and anxiety in general adult populations and among patients with an anxiety disorder [13], and in lowering depression, stress, and anxiety symptoms in students and young adults [14,15], and may be useful for other populations as well. A group that may benefit from stress-reduction through this non-invasive stress-reducing intervention is that of pregnant women.

Pregnancy is not only a source of joy, but may also include feelings of stress. During pregnancy, efforts to reduce or limit the level of stress appear to be highly relevant to lower associated mental health problems. Research has convincingly shown that high levels of maternal stress, anxiety, and depression during pregnancy are not only harmful for the woman herself, but may also affect the child she is carrying. For instance, poor maternal mood during pregnancy has been related to adverse birth outcomes, including preterm birth [16], difficult infant temperament [17,18], and adverse behavioral and developmental trajectories later in life [19,20,21]. A recent study showed that high levels of maternal trait anxiety and pregnancy-related anxiety during pregnancy also predicted more parenting stress in the first three months of life [22], suggesting that poor prenatal maternal mood may be carried over to more stress-related problems in the postpartum period. This combination of prenatal exposure to maternal anxiety and related high levels of maternal stress hormones [23,24] and postnatal exposure to parenting stress may hamper the optimal development of children and burden young parents at the same time.

For psychiatric disorders, such as major depression disorder or generalized anxiety disorder, women can receive psychiatric and psychological evidence-based treatment, such as cognitive behavioral therapy (CBT). However, fewer evidence-based interventions are available for less severe levels of stress, anxiety, and depression. This is alarming given that subclinical stress and related complaints can still negatively affect general well-being and productivity, and can be potentially harmful during pregnancy [25]. It is striking that relatively little research effort has been put into developing and testing interventions that have the potential to reduce maternal mental health problems during this important transitional life phase for women. The prenatal period in particular offers an important window of opportunity to optimize the circumstances of fetal development, and to implement interventions that may prevent adverse outcomes for both the mother and her child.

A few randomized controlled trials have been conducted to test the efficacy of treatment interventions on maternal antenatal mental health problems. In a systematic review, Fontein-Kuipers et al. [26] demonstrated a small but significant reduction in anxiety and depressive symptoms when pooling three trials using different stress-reducing techniques (i.e., a self-help support book, mindfulness training or acupuncture). HRV-biofeedback has also been tested in two studies for its effect on prenatal stress and birth outcomes [27,28], with positive results. Cullins et al. [27] compared a group of women with pregnancy-induced hypertension receiving HRV-biofeedback with a similar historical treatment-as-usual control group and found that gestational age was longer and birth weight at delivery was higher in the HRV-biofeedback group than in the control group. Siepmann et al. [28] did not find a statistically significant difference in preterm birth between a HRV-biofeedback condition and a control condition in women with preterm labor. However, their findings showed a reduction in perceived chronic stress after HRV-biofeedback. Thus, HRV-biofeedback seems to be a promising approach to alleviate pregnancy-related stress.

It is possible that the effect of HRV-biofeedback on mental health is different in pregnant versus non-pregnant women. HRV-biofeedback works on the cardiovascular system and pregnancy is characterized by physiological changes such as an increase in blood volume and peripheral vasodilatation [29]. Also, pregnancy-related higher cortisol levels and a blunted HPA-axis response to glucocorticoids [29] may affect the stress response and thereby the stress-reducing effect of HRV-biofeedback. Additionally, autonomic balance is altered during pregnancy, indicated by an increase in sympathetic activity of the autonomic nervous system and a decreased high-frequency HRV [30]. These differences between pregnant and non-pregnant women in the physiological stress response may also lead to differences in the effect of HRV-biofeedback on stress-related mental health. In order to examine these differences, this study focused on pregnant as well as on non-pregnant women.

HRV-biofeedback as a stress-reducing technique is still relatively new and well-controlled studies on the use of HRV-biofeedback as such are still scarce, as reviewed by Goessl et al. [13] in their meta-analysis. In our study, we therefore aimed to test whether HRV-biofeedback is effective in reducing health complaints, including stress, anxiety, and depression, and in improving general well-being and sleep quality, in both pregnant and non-pregnant women. Furthermore, we evaluated whether HRV-biofeedback has a different effect in pregnant women compared to non-pregnant women.

To inform future HRV-biofeedback programs, we examined whether total time spent on practicing HRV-biofeedback techniques mattered for its potential efficacy, as was suggested in a previous study of this group [15]. Reiner [31] also found a dose-response effect in patients with anxiety disorders, but not all studies have found such an effect [13]. Goessl et al. [13], however, based the HRV-biofeedback dose on the number of training sessions, while the former two studies used exercise time or points as a measure for HRV-biofeedback dose. To study the effect of practicing time on stress and related symptoms, we compared participants with high breathing exercise adherence to those with relatively low adherence. Furthermore, a dose–response analysis was carried out.

To answer our research questions, we conducted a quasi-experimental design, including an experimental and waitlist control condition. We compared a group of pregnant women and non-pregnant women on pre- and post-intervention self-report measures of stress, anxiety, depression, sleep, and psychological well-being. Based on previous research [13], we expected a stronger reduction in general stress, anxiety, and depression, and a stronger improvement of sleep and psychological well-being in the HRV-biofeedback condition than in the waitlist condition. Furthermore, we expected that higher breathing-exercise adherence would be associated with stronger improvements in the aforementioned aspects [15,31]. To the authors’ knowledge, there are no previous studies that compared the stress-reducing effect of HRV-biofeedback between pregnant and non-pregnant women. Therefore, these analyses were explorative in nature.

## 2. Materials and Methods

### 2.1. Participants and Recruitment

Participants were recruited via posters and flyers spread throughout Amsterdam, targeting both pregnant and non-pregnant women who suffered from stress. Furthermore, we posted messages on social media platforms and websites. Participants received the HRV-biofeedback training for free, and no other remuneration was provided for participation. Inclusion criteria were age between 18 and 40 years, having sufficient command of the Dutch language, and a score of 17 or higher on the 10-item Perceived Stress Scale (PSS) [32]. This cut-off score is 1 SD below the normative mean [33] and was chosen because we wanted to include people who suffered from stress, whether clinical or not. Importantly, we also wanted to ensure room for improvement. Exclusion criteria were chronic medical conditions such as Diabetes, use of medication known to affect cardiovascular stress reactivity measures (e.g., β-blockers), substance abuse, a score of 16 or higher on the depression scale of the HADS (cut-off score for severe depression; range 0–21) [34], and a history of major depressive disorder, bipolar disorder, or a psychotic disorder. All participants gave written informed consent. The Medical Ethical Committee of VU University Medical Center Amsterdam approved the study on 19 March 2014 (reference 13/360). This study is part of the PAIRS study, registered under clinical trial registration number NTR4599.

### 2.2. Experimental Procedures

**Figure 1:**
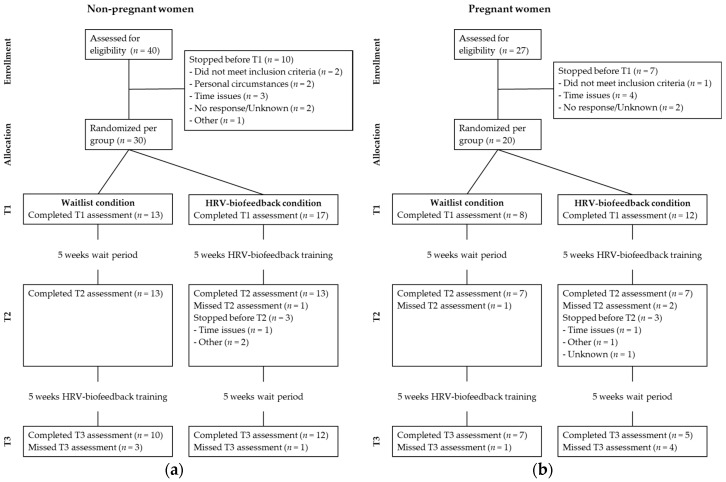
CONSORT flow diagram of participant flow through the study of: (**a**) non-pregnant participants; (**b**) and pregnant participants.

Data collection took place between June 2014 and September 2016. Participants were randomized to either an intervention condition or a waitlist condition. The intervention consisted of a HRV-biofeedback group-training. Randomization was performed at the group level and recruitment to fill a group was set to a maximum of 10 weeks. Consequently, each training group consisted of two to six women who received HRV-biofeedback training together, with a mixed composition of pregnant and non-pregnant women. Participants filled out a series of online questionnaires to measure demographics, stress and stress-related symptoms, including anxiety, depression, sleep quality and psychological well-being. In the intervention condition, all questionnaires were completed prior to (T1) and immediately after (T2) the HRV-biofeedback training, and six weeks later (T3). In the waitlist condition, all questionnaires were completed over a period with similar intervals, and participants in this condition received the HRV-biofeedback training between assessments T2 and T3 (see Figure 1). During the intervention period, participants kept a daily diary (on paper) reporting the duration of and experience with their paced breathing exercises.

### 2.3. Outcome Measures

We used the Dutch version of the Depression Anxiety Stress Scales (DASS) [35] to measure depression, anxiety, and stress over the previous week. The DASS-21 consists of three seven-item subscales: (a) depression, (b) anxiety, and (c) stress. Items were rated on a four-point Likert scale, with response options ranging from 0 (did not apply to me at all) to 3 (applied to me very much, or most of the time). Higher scores indicated higher levels of depressive symptoms, anxiety, or stress. Internal consistency was sufficient to good (Cronbach’s α was 0.86 for Depression, 0.75 for Anxiety and 0.83 for Stress at T1).

We measured sleep quality with the Dutch version of the Pittsburgh Sleep Quality Index (PSQI) [36]. The PSQI contains 19 items on sleep during the past month, addressing seven components of sleep: (a) sleep quality, (b) sleep latency, (c) sleep duration, (d) habitual sleep efficiency, (e) sleep disturbances, (f) use of sleeping medication, and (g) daytime dysfunction. A score of 0 to 3 is given for each component after which a sum score is calculated. A sum score >5 represents poor sleep. Based on the component scores, Cronbach’s α at T1 was good (0.80).

We assessed psychological well-being using the 39-item Dutch version of the Scales of Psychological Well-being (SPW) [37,38]. Participants rated statements about well-being on a six-point Likert scale, ranging from 1 (totally disagree) to 6 (totally agree) with higher scores indicating higher levels of psychological well-being. Internal consistency in the present sample was very good (Cronbach’s α at T1 was 0.93).

### 2.4. Stress-Reducing Intervention

The stress-reducing intervention consisted of 5 weekly meetings of 60 to 90 min duration each. The training was given by two trainers per intervention group according to a protocol [39], which was based on the HRV-biofeedback protocol developed by Lehrer, Vaschillo and Vaschillo [40]. It also included common stress-management techniques, such as psychoeducation and weekly assignments in which participants had to register stress levels and well-being. During the training, psychoeducation about stress and stress responses and about HRV-biofeedback was provided, and participants were taught abdominal breathing and HRV-biofeedback. Participants were instructed to do daily breathing exercises at home increasing in duration over time: week 1: 10 min/day, week 2: 15 min/day, week 3: 2 × 15 min/day, and weeks 4 and 5: 2 × 20 min/day. Furthermore, participants received behavioral exercises such as registering complaints and experimenting with stress-reducing strategies, for example realistically planning next week’s obligations and leisure activities. Treatment integrity was monitored using self-reported adherence to protocol instructions after each session. Each session consisted of 7 to 9 elements (e.g., psychoeducation on benefits of HRV-biofeedback), with 43 elements in total over five sessions. If an element was not covered in one session, it was added to the next session when possible. Treatment integrity was very high as 95% of all elements were covered during the interventions. If a participant could not attend one of the sessions, she received a short (approximately 30 min) private session in person or over the phone.

### 2.5. HRV-Biofeedback

Prior to the training, a personal resonance frequency was determined for each participant, i.e., the breathing frequency at which HRV is maximal [41]. Heart rate and respiration rate were recorded using the NeXus-4 hardware (NeXus-4, Mind Media, Herten, The Netherlands) and BioTrace+ software (BioTrace+ for Nexus-4, version 2013, Mind Media, Herten, The Netherlands). Electrodes were placed on the wrists with a reference electrode on the left forearm [42]. Respiration rate was recorded using a respiration belt around the torso. Following the protocol of Lehrer, Vaschillo and Vaschillo [40], participants breathed in a pace of 6.5, 6.0, 5.5, 5.0, and 4.5 breaths per minute for several minutes each, using a pacer. After a 10 min break, the three frequencies resulting in the highest HRV were repeated, followed by a biofeedback trial in which the participants followed the variation in their heart rate with their respiration. The frequency that resulted in the highest HRV was considered to be the resonance frequency.

Participants used the StressEraser (a 510 [k] premarket notification-exempt, class II medical device; Helicor, New York) for the daily exercises. This non-invasive hand-held device uses an infrared finger photoplethysmograph to measure inter-beat-intervals in the pulse rate, which are used for assessing respiratory sinus arrhythmia. When practicing with the StressEraser, participants tried to increase their HRV by breathing at their resonance frequency.

### 2.6. Treatment Adherence

During the five-week intervention, participants recorded daily whether and for how long they had conducted the paced breathing exercises. In order to maximize adherence, an implementation plan was made during each training session in which participants scheduled a time and place for each of the daily exercises in the upcoming week.

### 2.7. Statistical Analyses

To test change over time the difference between T1 and T2 in the outcome measures was evaluated with paired Student’s *t* tests for both conditions separately, irrespective of pregnancy. Additionally, the effect of condition was examined using multiple regression analysis with the outcome measures at T2 as dependent variables, condition as an independent variable and the outcome measure at T1 as a covariate, to control for regression to the mean. In order to test whether the treatment effect differed between pregnant and non-pregnant women, the variables Pregnancy and the Condition–Pregnancy interaction were added to these multiple regressions.

Missing data were handled according to the guidelines of the Committee for Medicinal Products for Human use [43] using multiple imputation (MI) with five imputation sets in the main analyses. All predictors and outcome variables were included in the imputation process. Performing analyses with a MI dataset with a small sample size could result in inflated degrees of freedom, therefore the correction suggested by Barnard and Rubin [44] was applied to the paired Student’s *t* tests that used the MI data set.

We performed two sensitivity analyses. First, in order to test the treatment efficacy in case of potential selective drop-outs, analyses were carried out using last observation carried forward (LOCF) imputations for data lost to follow-up. Second, in order to estimate an optimized treatment effect, the analyses were carried out only on participants with a total exercise duration of 400 min or more in the HRV-biofeedback condition. A 400-min training effort has been demonstrated to result in significant stress and anxiety reduction [14], which is about half of the exercise time in our training protocol.

Finally, we examined whether the time spent on exercises was related to the overall efficacy (dose–response relationship) in the HRV-biofeedback condition, using multiple regression with the outcome measures at T2 as dependent variables, exercise time as independent variable, and the outcome measure at T1 as a covariate.

All analyses were conducted in SPSS version 23.0 and *p* values < 0.05 were considered statistically significant.

## 3. Results

### 3.1. Sample Characteristics

Demographic characteristics of the sample are shown in Table 1. The randomization procedure led to more participants in the HRV-biofeedback condition than in the waitlist condition (58% and 42%, respectively). There were no significant differences between conditions in age, level of education, work, or pregnancy or, for pregnant women, in parity (the number of pregnancies carried to a viable gestational age) or gestational age (all *p* values > 0.05). Outcome variables at T1 did not differ between conditions (all *p* values > 0.05), with the exception of the DASS depression subscale (*T*(27.13) = 2.15, *p* = 0.040). Participants in the waitlist condition scored somewhat higher on depression than those in the HRV-biofeedback condition (means were 9.81 (*SD* = 8.55) and 5.45 (*SD* = 4.42), respectively).

There were no significant differences between dropouts and non-dropouts for age, level of education, work, pregnancy, parity, gestational age, depression, anxiety, stress, or sleep (all *p* values > 0.05). However, there were fewer dropouts in the waitlist condition than in the HRV-biofeedback condition (*n* = 1 and *n* = 9, respectively; *χ*^2^(1) = 5.26, *p* = 0.022), and on average, dropouts scored lower on psychological well-being than non-dropouts (means were 145.90 (*SD* = 19.36) and 163.90 (*SD* = 22.81, respectively); *T*(48) = -2.29, *p* = 0.026).

### 3.2. Change over Time

Table 2 shows the means and standard deviations of the outcome variables in the waitlist and HRV-biofeedback conditions at each assessment and the associated effect sizes over time. In both conditions, depression, anxiety, stress, and poor sleep levels were reduced and well-being increased between pre- and post-test (T1–T2). In the HRV-biofeedback condition, within-group effect sizes were medium [45], and long-term improvements six weeks after the training (T1–T3) were similar to those at post-test for all outcome measures except depression. Statistically significant long-term improvements in the HRV-biofeedback condition were present for stress and psychological well-being.

Effect sizes were larger in the HRV-biofeedback condition than in the waitlist condition on all outcome variables except anxiety (see Table 2). Psychological well-being improved significantly more in the HRV-biofeedback condition than in the waitlist condition (see Table 3, left part). In other words, we found a beneficial effect of HRV-biofeedback on psychological well-being.

### 3.3. Pregnant and Non-Pregnant Women

**Figure 2:**
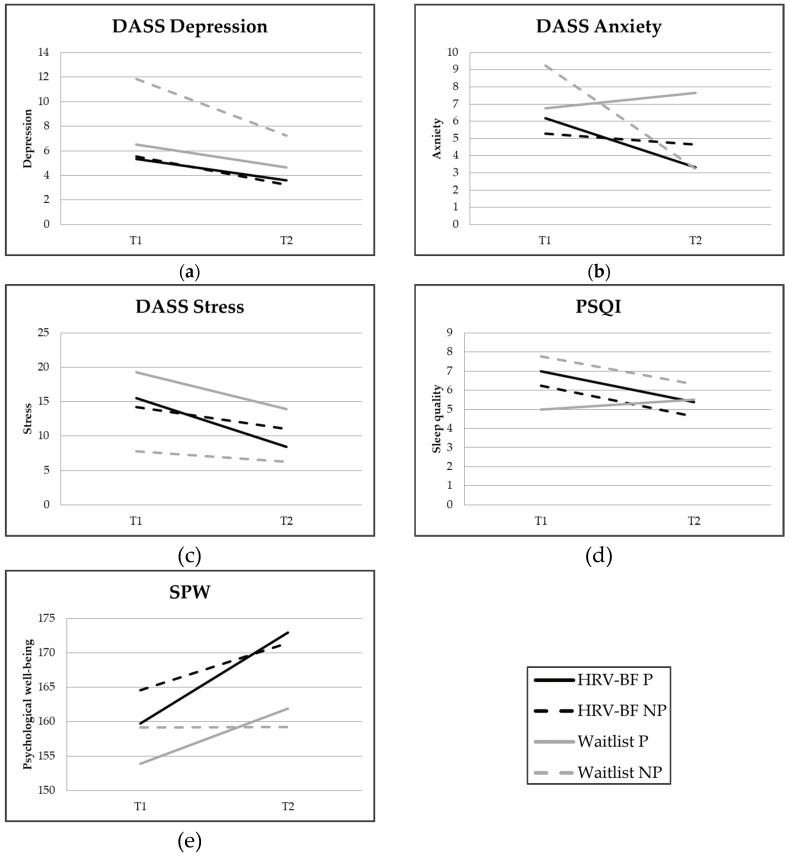
Mean scores on the (**a**) Depression scale of the Depression Anxiety Stress Scale (DASS); (**b**) Anxiety scale of the DASS; (**c**) Stress scale of the DASS; (**d**) Pittsburgh Sleep Quality Index (PSQI); (**e**) Scales of Psychological Well-being (SPW) per intervention condition for pregnant and non-pregnant women at pre-test (T1) and post-test (T2). Note: a higher PSQI score represents poorer sleep quality.

Figure 2 shows the change over time of the outcome variables in the waitlist and HRV-biofeedback conditions for both pregnant and non-pregnant women. When comparing the treatment effect between pregnant and non-pregnant women (the Condition–Pregnancy interaction), a statistically significant interaction effect for anxiety appeared (Table 3, right part). Additional analyses showed that HRV-biofeedback was more beneficial regarding anxiety reduction for pregnant women than for non-pregnant women (pregnant women: *B* = −4.18, *t* = −2.74, *p* = 0.006; non-pregnant women: *B* = 2.55, *t* = 1.99, *p* = 0.046).

### 3.4. Sensitivity Analyses

In addition to the MI method used for the imputation of missing data, we used the more conservative last observation carried forward (LOCF) method [43] for the change over time, to assess the treatment efficacy in case of potential selective dropout due to unobserved data at T2. One participant in the waitlist condition dropped out and nine participants did so in the HRV-biofeedback condition. In Table 4 (left part), the results for the HRV-biofeedback condition using LOCF imputation are shown (for the waitlist condition, the results were similar to the results in Table 2, since only one participant dropped out). As expected, the effect sizes were smaller when using LOCF imputation, but the change in scores of the DASS Stress was still statistically significant for both time periods, as was the long-term change in scores on the SPW.

Conditions did not differ in change over time for any of the outcome variables when using LOCF imputation (all *p* values > 0.05). Furthermore, when comparing pregnant and non-pregnant women, the beneficial effect of HRV-biofeedback on anxiety remained significantly higher in pregnant women compared to non-pregnant women (Condition × Pregnancy: *B* = −5.83, *SE* = 2.03, *t* = −2.88, *p* = 0.006; pregnant women: *B* = −3.33, *SE* = 1.56, *t* = −2.13, *p* = 0.039; non-pregnant women: *B* = 2.50, *SE* = 1.30, *t* = 1.93, *p* = 0.060).

To assess an optimized training effect, based on the guidelines of Henriques et al. [14] we selected women who reported at least 400 min of training (high training subsample; *n* = 13), and compared this group with those in the waitlist condition. Table 4 (right part) shows the short-term and long-term effect sizes for the high training subsample. None of the women selected for >400 min of training were missing data at T3; therefore, we used the original non-imputed dataset for the analyses of the long-term change over time (Table 4, right part, T1–T3). Unexpectedly, effect sizes in the high training subsample were lower than in the total HRV-biofeedback condition. We did not find significant differences between the high training subsample and the waitlist condition in the regression analyses for any of the outcome variables (all *p* values > 0.05).

### 3.5. Adherence

Twenty-four of the 29 women in the HRV-biofeedback condition returned the daily diaries with exercise information. On average, these participants performed breathing exercises for 455.56 min (*SD* = 255.23; range 69 min to 894 min). We did not find an association between exercise duration and any of the outcome variables (all *p* values > 0.05).

## 4. Discussion

The goal of this study was twofold, namely (1) to assess the efficacy of HRV-biofeedback in reducing stress, anxiety, and depression, and in improving general well-being and sleep quality among women, and (2) to assess whether the efficacy differed between pregnant and non-pregnant women. At post-test, women in both the HRV-biofeedback and the waitlist conditions showed fewer complaints of stress and depression, and women in the HRV-biofeedback condition reported improved psychological well-being. No statistically significant effects were found on sleep and anxiety for both groups. Women in the HRV-biofeedback condition improved significantly more on psychological well-being than women in the waitlist condition. This effect was robust, as the effect remained significant after re-analysis using a conservatively imputed data-set using the LOCF method [43]. Moreover, the improvements with respect to stress and well-being in the HRV-biofeedback condition were maintained at follow-up, six weeks after post-test. With respect to the differences in efficacy of HRV-biofeedback in pregnant and non-pregnant women, we found that HRV-biofeedback resulted in a larger significant reduction in anxiety among pregnant women than among non-pregnant women. This difference in treatment effect was also robust; it remained significant when using the more conservative imputation method. In sum, HRV-biofeedback had a significant positive effect on psychological well-being for all women and had an additional significant beneficial effect on anxiety complaints for pregnant women.

Our findings that HRV-biofeedback had a beneficial effect on stress and depressive symptoms are in line with previous studies among non-clinical populations of manufacturing operators [46] and young adults [15]. We did not replicate the beneficial treatment effect on anxiety of other studies among healthy psychology and nursing students [14,47] in the total group. However, we did find an effect of HRV-biofeedback on anxiety in the group of pregnant women. A possible explanation for this difference is that the total HRV-biofeedback group in our study had low baseline scores on anxiety, with *M* = 5.66 at T1, while the cut-off score for mild anxiety is 8 [48]. This left little room for improvement. However, for pregnant women, anxiety tends to increase throughout pregnancy, potentially creating more room for improvement. This explanation is supported by the finding that anxiety increased in pregnant women in the waitlist group (means were 6.75 and 7.65 at T1 and T2, respectively). Finally, our findings that a HRV-biofeedback training did not improve sleep quality, but did improve psychological well-being were in line with previous findings in an effect study in a group of healthy young adults [15].

Unexpectedly, we found no evidence for a larger treatment effect among participants who spent a substantial amount of time on HRV-biofeedback breathing exercises. This finding thus suggests that, for women from the general population reporting mild to severe stress complaints, it is possible to get an optimal treatment effect even if one has not spent 400 min on breathing exercises yet. Increasing treatment adherence thus may not result in additional improvement. This is supported by our finding that there was no dose–response relationship between breathing exercise time and any of the outcome variables. These results are in line with the meta-analysis on the effect of HRV-biofeedback training of Goessl et al. [13]. This meta-analysis reported no association between the number of training sessions and the effect of HRV-biofeedback on stress. Thus, our findings provide further evidence for the observation that HRV-biofeedback may already be effective after short exercise. However, caution is warranted with this interpretation, as also other factors may influence the association between exercise time and the effect of HRV-biofeedback, such as the severity of mental health complaints. To illustrate, in our sample from the general population, there was a negative correlation between stress level at baseline and exercise duration (*r*(22) = −0.43, *p* = 0.03), suggesting that individuals with more severe complaints practiced less, but had more room for improvement of their well-being. Hence, a floor effect may be partly responsible for our results on the dose–response relationship between training exercise and health improvement. Therefore, more research is required to examine the amount of exercise time that brings about the best health improvement.

### 4.1. Limitations and Strengths

A limitation of this study was the lack of a control condition for the follow-up measurement, because the waitlist condition received HRV-biofeedback training in between assessment waves two and three. Therefore, we could not study long-term treatment effects. However, if we had let pregnant women in the waitlist condition wait for the training until after the third assessment wave, they might not have been able to complete the training before giving birth. Another limitation is that the size of our subsamples for some of the analyses was quite small (e.g., *n* = 13 for the HRV-biofeedback sample with high adherence that was used in the sensitivity analyses) and, therefore, small effects may have gone undetected due to insufficient statistical power. Furthermore, participants with a high education level were overrepresented in our sample, and thus results may not be generalizable to populations with a lower education level. A possible limitation is that the researchers who conducted the study were not blinded to the intervention condition of the participants. However, since participants filled out the questionnaires at home and they were aware that data were stored anonymously, we consider the risk that researchers affected the answers of the participants to be very low, despite this knowledge. Finally, it is possible that our findings are not only the result of the HRV-biofeedback training, but also of the more common stress-management techniques that were applied in our intervention. However, our findings are in line with other studies that only used HRV-biofeedback as a stress-reducing technique [14]; hence, it is likely that HRV-biofeedback at least partly contributes to stress-reduction.

A strength of our study is that the size of the total sample was relatively large for a HRV-biofeedback study. Furthermore, an individual resonance frequency was determined for each participant providing participants with the right breathing frequency for obtaining a large HRV. In this way, participants could use their personalized optimal breathing frequency during exercises and validity of the HRV-biofeedback intervention was secured.

### 4.2. Future Research

In addition to the improvement of psychological well-being, several trends were visible in the data of the current study (e.g., sleep), suggesting that HRV-biofeedback training could promote relevant albeit small improvements in five weeks’ time. Therefore, it is worthwhile for future research to replicate the study with larger sample sizes. Future research could also examine whether there is a threshold rather than a dose-response effect with respect to exercise time for HRV-biofeedback to be effective, since various studies suggest that a low ‘dose’ might already have an effect [13,49,50]. Finally, because there seems to be a different effect of HRV-biofeedback in pregnant women compared to non-pregnant women, it might be worthwhile to study the former group separately. This would allow for more focus on the effects of HRV-biofeedback on pregnancy-related aspects, such as pregnancy-specific anxiety and birth outcomes, as well as effects on the child.

### 4.3. Clinical Implications

In line with the findings reported in a recent meta-analysis [13], the beneficial effect of HRV-biofeedback we found in our study further supports using this technique among women reporting stress and related complaints in clinical practice. Further research into combining HRV-biofeedback with more cognitive and behaviorally oriented treatment modules is indicated, to assess whether treatment benefits can be increased. This study also provides additional support for use of HRV-biofeedback as an anxiety-reducing technique during pregnancy, as in our study HRV-biofeedback resulted in a reduction of anxiety, while the pregnant women in the control group reported an increase of anxiety, which is commonly found during pregnancy [51,52,53]. Additionally, we found that the technique can be mastered in a relatively short time, also by pregnant women, which was demonstrated by Cullins et al. [27] as well.

## 5. Conclusions

In conclusion, HRV-biofeedback is a low-cost, low-threshold technique that improves well-being among women reporting stress, and results in less anxiety among pregnant women. Future research may focus on whether HRV-biofeedback aids in limiting adverse consequences of maternal anxiety during pregnancy on her offspring.

## Figures and Tables

**Table 1:** Demographic characteristics of participants in the waitlist and heart rate variability biofeedback (HRV-BF) condition at T1.

Characteristics	Total	Waitlist	HRV-BF	*T* or *χ*^2^	*p* Value	Effect Size
	*N* = 50	*n* = 21	*n* = 29			
Age (years) (mean, *SD*)	31.6 (5.9)	31.3 (6.1)	31.8 (5.9)	*T*(48) = −0.31	0.760	0.09 ^3^
Level of education (*n*, %)				*χ*^2^(3) = 3.29	0.349	0.15 ^2^
High school	6 (12.0)	2 (9.5)	4 (13.8)			
Lower vocational school	3 (6.0)	-	3 (10.3)			
Higher vocational school	11 (22.0)	4 (19.0)	7 (24.1)			
University	30 (60.0)	15 (71.4)	15 (51.7)			
Work (*n*, %)				*χ*^2^(3) = 5.43	0.143	0.19 ^2^
College	9 (18.0)	5 (23.8)	4 (13.8)			
Full-time	14 (28.0)	7 (33.3)	7 (24.1)			
Part-time	17 (34.0)	8 (38.1)	9 (31.0)			
Unemployed/On leave	10 (20.0)	1 (4.8)	9 (31.0)			
Pregnancy (*n*, %)				*χ*^2^(1) = 0.06	0.815	0.03 ^2^
Non-pregnant	30 (60.0)	13 (61.9)	17 (58.6)			
Pregnant	20 (40.0)	8 (38.1)	12 (41.4)			
Parity (*n*, % of pregnant)				*χ*^2^(1) = 2.06	0.152	0.17 ^2^
Nulliparous	13 (65.0)	6 (75.0)	7 (58.3)			
Primiparous or multiparous	7 (35.0)	2 (25.0)	5 (41.7)			
GA ^1^ at T1 (mean, *SD*)	19.33 (5.2)	21.0 (4.9)	18.2 (5.3)	*T*(18) = 1.2	0.243	0.56 ^3^

^1^ GA = Gestational age in weeks; ^2^ Cohen’s *d*; ^3^ Cramer’s *V*.

**Table 2:** Observed means and Cohen’s *d* within-group effect sizes for stress and stress-related symptoms per condition.

Measure	Condition	T1		T2		T3 ^1^		T1–T2			T1–T3		
		*M*	*SD*	*M*	*SD*	*M*	*SD*	*T(df)*	*p*	*d*	*T(df)*	*p*	*d*
DASS Depr.	Waitlist	9.81	8.55	6.40	4.97	6.12	8.20	−2.45 (19.21)	0.024	−0.46			
	HRV-BF	5.45	4.24	2.80	3.40	4.22	3.56	−2.21 (19.20)	0.039	−0.47	−1.00 (25.05)	0.327	−0.23
DASS Anx.	Waitlist	8.29	8.30	4.90	4.66	6.12	6.50	−2.07 (19.08)	0.053	−0.46			
	HRV-BF	5.66	4.28	4.10	4.08	3.89	4.57	−1.71 (24.75)	0.101	−0.39	−1.26 (22.74)	0.220	−0.30
DASS Stress	Waitlist	17.71	9.26	13.00	6.76	12.82	9.06	−3.06 (18.81)	0.007	−0.55			
	HRV-BF	14.76	5.33	9.60	6.24	9.56	7.18	−4.52 (24.45)	<0.001	−0.85	−3.13 (20.99)	0.005	−0.64
PSQI	Waitlist	6.71	3.21	6.05	2.63	5.50	2.71	−1.48 (18.23)	0.156	−0.23			
	HRV-BF	6.55	4.15	4.70	2.66	4.20	1.93	−1.98 (18.17)	0.063	−0.38	−1.87 (9.98)	0.091	−0.37
SPW	Waitlist	157.14	26.04	160.10	22.39	164.59	20.92	1.11 (18.53)	0.283	0.12			
	HRV-BF	162.59	20.98	174.25	17.12	171.28	15.72	2.52 (12.60)	0.026	0.38	2.65 (24.70)	0.014	0.40

Anx.: Anxiety, DASS: Depression Anxiety Stress Scale, Depr.: Depression, *df*: degrees of freedom, HRV-BF: heart rate variability biofeedback, *M*: mean, PSQI: Pittsburgh Sleep Quality Index, *SD*: standard deviation, SPW: Scales of Psychological Well-being; ^1^ T3 is the post-intervention measurement for the waitlist group, and the follow-up assessment for the HRV-biofeedback group.

**Table 3:** Multiple regression analyses for stress and stress-related symptoms at T2, condition, and pregnancy.

Predictors	Condition				Condition and Pregnancy			
	*B*	*SE*	*t*	*p*	*B*	*SE*	*t*	*p*
DASS Depression	−1.40	1.08	−1.30	0.193				
Condition					−1.96	1.39	−1.40	0.160
Pregnancy					−0.86	1.63	−0.52	0.600
Condition × Pregnancy					1.28	2.14	0.60	0.548
DASS Anxiety	−0.16	1.10	−0.15	0.881				
Condition					2.55	1.28	1.99	**0.046**
Pregnancy					5.13	1.55	3.31	**0.001**
Condition × Pregnancy					−6.73	1.97	−3.42	**0.001**
DASS Stress	−1.57	1.47	−1.07	0.286				
Condition					−0.03	1.81	−0.01	0.989
Pregnancy					0.46	2.20	0.21	0.836
Condition × Pregnancy					−3.70	2.87	−1.29	0.197
SPW	8.48	3.26	2.60	**0.009**				
Condition					8.81	4.17	2.11	0.035
Pregnancy					5.98	5.04	1.19	0.235
Condition × Pregnancy					−1.44	6.78	−0.21	0.832
PSQI	−1.00	0.60	−1.69	0.092				
Condition					−1.10	0.81	−1.36	0.173
Pregnancy					0.23	1.06	0.21	0.831
Condition × Pregnancy					0.22	1.33	0.17	0.866

Statistically significant results concerning hypothesized effects are depicted in bold; DASS: Depression Anxiety Stress Scale, PSQI: Pittsburgh Sleep Quality Index, SPW: Scales of Psychological Well-being.

**Table 4:** Cohen’s *d* within-group effect sizes for stress and stress-related symptoms for participants in the HRV-biofeedback condition.

Measure	Last Observation Carried Forward						High Training Subsample (Training Duration >400 min)					
	T1–T2			T1–T3			T1–T2			T1–T3		
	*T(df)*	*p*	*d*	*T(df)*	*p*	*d*	*T(df)*	*p*	*d*	*T(df)*	*p*	*d*
DASS Depression	−1.82 (28)	0.080	−0.21	−1.10 (28)	0.279	−0.15	−1.73 (9.61)	0.116	−0.49	−0.49 (12)	0.632	−0.15
DASS Anxiety	−1.71 (28)	0.098	−0.27	−1.57 (28)	0.127	−0.31	−0.26 (10.20)	0.802	−0.07	−0.27 (12)	0.788	−0.11
DASS Stress	−3.83 (28)	**0.001**	−0.46	−3.19 (28)	**0.004**	−0.46	−2.15 (10.07)	0.057	−0.62	−1.05 (12)	0.315	−0.26
PSQI	−1.35 (28)	0.188	−0.14	−1.05 (28)	0.301	−0.11	−0.54 (10.17)	0.598	−0.16	−0.50 (11)	0.626	−0.15
SPW	1.98 (28)	0.058	0.14	2.42 (28)	**0.022**	0.23	1.02 (9.86)	0.334	0.18	0.63 (12)	0.543	−0.11

Statistically significant results concerning hypothesized effects are depicted in bold; DASS: Depression Anxiety Stress Scale, *df*: degrees of freedom, HRV-BF: heart rate variability biofeedback, PSQI: Pittsburgh Sleep Quality Index, SPW: Scales of Psychological Well-being.
